# Pattern of presenting complaints recorded as near-drowning events in emergency departments: a national surveillance study from Pakistan

**DOI:** 10.1186/1471-227X-15-S2-S4

**Published:** 2015-12-11

**Authors:** Siran He, Jeffrey C Lunnen, Nukhba Zia, Uzma Rahim Khan, Khusro Shamim, Adnan A Hyder

**Affiliations:** 1 Johns Hopkins International Injury Research Unit, Department of International Health, Johns Hopkins Bloomberg School of Public Health, Baltimore, MD, USA; 2 Department of Emergency Medicine, Aga Khan University, Karachi, Pakistan

**Keywords:** Drowning, near-drowning, emergency care management, Pakistan

## Abstract

**Background:**

Drowning is a heavy burden on the health systems of many countries, including Pakistan. To date, no effective large-scale surveillance has been in place to estimate rates of drowning and near-drowning in Pakistan. The Pakistan National Emergency Department Surveillance (Pak-NEDS) study aimed to fill this gap.

**Methods:**

Patients who presented with a complaint of "near-drowning" were analyzed to explore patterns of true near-drowning (unintentional) and intentional injuries that led to the "near-drowning" complaint. Bivariate analysis was done to establish patterns among patients treated in emergency departments, including socio-demographic information, injury-related information, accompanying injuries, and emergency department resource utilization.

**Results:**

A total of 133 patients (0.2% of all injury patients) with "near-drowning" as presenting complaints were recorded by the Pak-NEDS system. True near-drowning (50.0%) and intentional injuries that led to "near-drowning" complaints (50.0%) differed in nature of injuries. The highest proportion of true near-drowning incidents occurred among patients aged between 25-44 years (47.5%), and among males (77.5%). True near-drowning patients usually had other accompanying complaints, such as lower limb injury (40.0%). Very few patients were transported by ambulance (5.0%), and triage was done for 15% of patients. Eleven (27.5%) true near-drowning patients received cardiopulmonary resuscitation.

**Conclusion:**

There was major under-reporting of drowning and near-drowning cases in the surveillance study. The etiology of near-drowning cases should be further studied. Patients who experienced non-fatal drownings were more commonly sent for medical care due to other accompanying conditions, rather than near-drowning event itself. There is also need for recognizing true near-drowning incidents. The results of this study provide information on data source selection, site location, emergency care standardization, and multi-sector collaboration for future drowning prevention studies.

## Background

Injuries are a large, yet often neglected, burden on health systems around the world [1]. The major types of injuries, such as road traffic injuries, falls, drowning, burns, poisoning, violence, and self-harm cause 10% of the total annual deaths, or 5 million lives lost globally [1,2]. Among all these causes, drowning caused 349,120 deaths in 2010, accounting for 7% of all injury-related deaths, with a drowning rate of 11.14 per 100,000 population [3,4]. The total number of drowning deaths increased to approximately 372,000 in 2014 [5]. Additionally, the burden of drowning deaths was disproportionately higher in certain regions in the world; for example, South Asia (Afghanistan, Bangladesh, Bhutan, India, Nepal and Pakistan) accounted for 122,957 drowning deaths - the highest number in 2010 compared to rest of the regions [3,5]. The death rate was 7.63 deaths per 100,000 population in this region in 2010. Effective drowning prevention work in this region could avert seven million disability-adjusted life years (DALYs) [3]. Despite these high rates, the actual drowning burden may have been significantly underestimated [1,4].

Drowning is defined by the World Health Organization (WHO) as "the process of experiencing respiratory impairment from submersion/immersion in liquid" [5]. Based on the outcome, the events are classified as either drowning (fatal drowning, which leads to death) or near-drowning (non-fatal drowning, which leads to morbidity or no morbidity) [4,6]. Children, males, and individuals with easy access to bodies of water have a higher risk of drowning [4]. Furthermore, in countries such as the United States, drowning affects more economically active individuals in a community, thus having many societal impacts that are both negative and difficult to measure [4,7]. Although near-drowning events do not result in death, they can leave the survivor temporarily unconscious, functionally impaired, mentally traumatized, or even permanently disabled, which may result in considerable direct and indirect financial costs [8]. These costs are much higher for those with severe chronic sequelae. For families and communities in low- and middle-income countries (LMICs), the financial repercussions of a drowning or near-drowning event are severe, especially when the injured person was relied upon for income-generating activities [9]. It is therefore equally important to detect near-drowning events. Death can be prevented if near-drowning cases are managed properly and drowning risk factors can be identified through the investigation of individual near-drowning cases [8].

Pakistan is the sixth most populous country in the world with a total population of 184 million [10]. It also has a higher rate of drowning (6.54 deaths per 100,000 population) than most other countries in South Asia [11]. A study from pre-hospital emergency medical services (EMS) in Punjab Province found that 3% of all "major incidents" were due to drowning [12]. Similarly, for those under 15 years of age, drowning was one of the main causes of injuries (3%) and a major cause of death (18%), with around half of all drowning incidents taking place in the sea [13]. A recent study also showed that drowning was the most common cause of death amongst children under the age of five years [14].

To date, no large-scale systematic data on drowning and near-drowning have been collected in low-resource environments, including Pakistan [7]. All estimations have been based on small-scale cross-sectional surveys and unsystematic data collection through primary health care systems. Emergency departments (EDs) function not only as an acute response system for emergency health conditions, but also as a frontline disease- recording system, especially in LMICs [15,16]. In countries where primary health care systems are fragmented and higher-level health care is cost-prohibitive, patients rely on EDs to address a wide variety of medical conditions [17]. Pakistan has extremely high needs for trauma and injury care and emergency services [16,18]. Nevertheless, the poor EMS infrastructure within the country has caused general dissatisfaction [19]. This article is one in a series of articles that carefully dissect information gathered through the larger Pakistan National Emergency Department Surveillance (Pak-NEDS) study. The objectives of this paper are: 1) to report the pattern of presenting complaints that were recorded as near-drowning events from this large-scale ED surveillance; 2) to compare true near-drowning events (unintentional) with intentional injuries that were incorrectly recorded as near-drowning but were later recognized otherwise; and 3) to highlight the urgent need to standardize ED surveillance systems for accurate injury data collection, especially for drowning and near-drowning incidents.

## Methods

**Figure 1:**
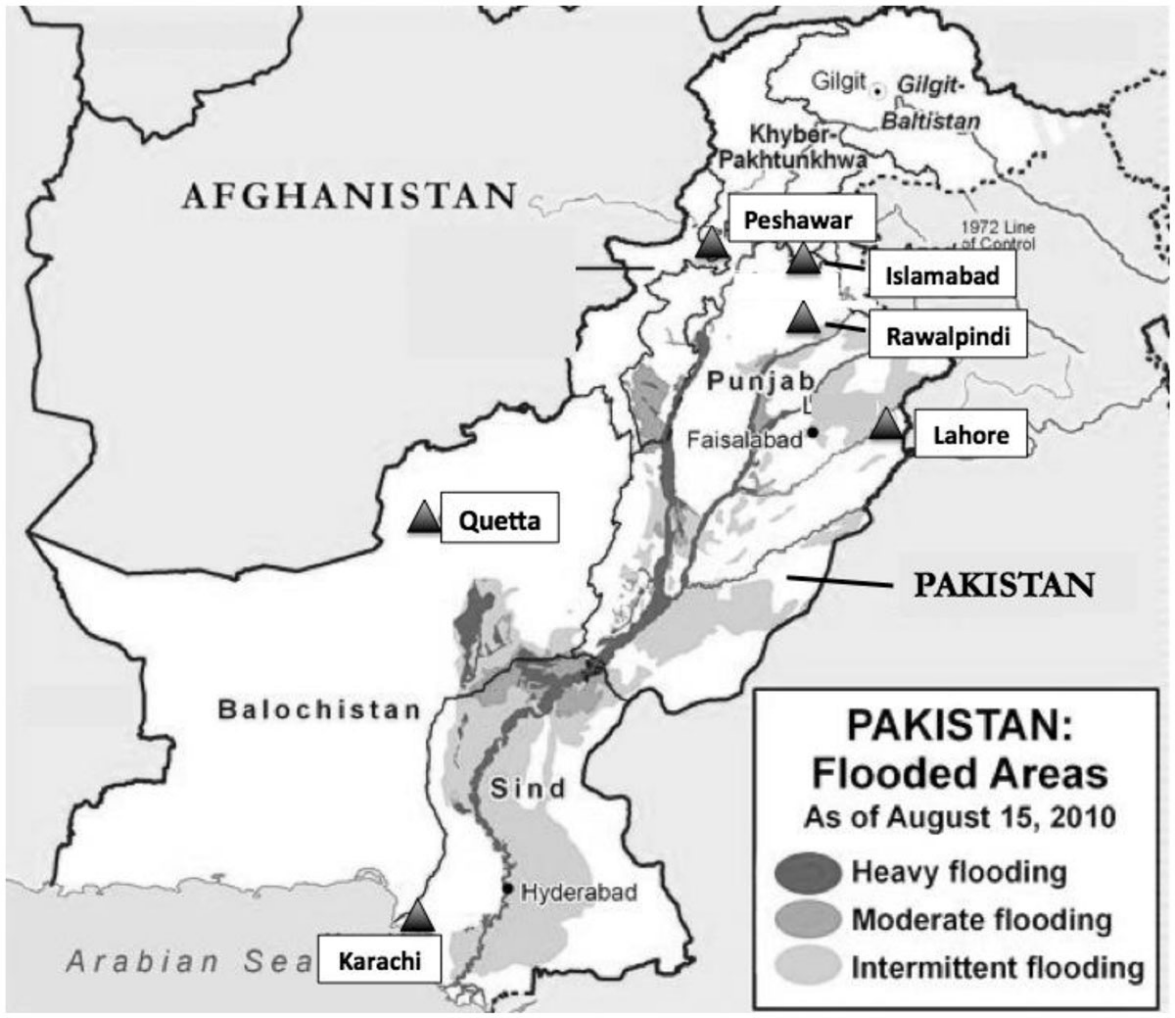
**Site of Hospitals in Pakistan National Emergency Department Surveillance Study and Pakistan Flood Areas in 2010 **[20]. Note: Black triangle - location of participating hospitals.

The Pak-NEDS study is an active pilot surveillance study that was conducted between November 2010 and March 2011 in seven urban tertiary care centers situated in five major Pakistani cities of five provinces. The seven study sites are in: 1) Sindh province - Aga Khan University and Jinnah Post-graduate Medical Center in Karachi; 2) Balochistan province - Sandeman Provincial Hospital in Quetta; 3) Punjab province - Benazir Bhutto Hospital in Rawalpindi, and Mayo Hospital in Lahore; 4) Islamabad Federal Capital Area - Shifa International Hospital in Islamabad; and 5) Khyber Pakhtunkhwa - Lady Reading Hospital in Peshawar. Figure 1 shows the locations of each study site in Pakistan and flood areas in 2010 [20].

Aga Khan University (AKU) served as the main coordinating center for the Pak-NEDS study. Ethical approval for this study was secured from a research ethics committee in each participating hospital, the AKU Ethical Review Committee and Institutional Review Board at the Johns Hopkins Bloomberg School of Public Health in the United States.

The Pak-NEDS tool was based on the Centers for Disease Control and Prevention, USA ambulatory care survey tool and previous work undertaken by the investigators [21,22]. The finalized Pak-NEDS tool is a standardized, one-page questionnaire that can be easily administered. The questionnaire includes identifiers (hospital ID, study ID) and major surveillance questions such as socio-demographic information (age, sex, ethnicity); main presenting complaint at ED; history of ED visits; types of diseases and injuries; initial triage; care providers seen at the visit; diagnostic/screening services and procedures; doctor's provisional diagnosis for the visit; disposition, and approximation of cost. The Pak-NEDS data collection tool allowed multiple responses for variables like "main complaints" which could be accompanied by up to two other complaints. Similarly, for "diagnostic/screening services," multiple choices could be selected to more accurately reflect ED services provided to patients.

All patients (both sexes across all age groups) who presented at the participating EDs were registered in the surveillance system. The study aims to establish a standardized active surveillance system across seven sites, therefore no exclusion criteria were employed. Over the four-month data collection period, trained data collectors were responsible for collecting data 24 hours a day, seven days a week in three shifts. The main sources of data were interviews with patients or their next of kin, and ED records. Data collection did not continue beyond the EDs; rather it ended with any actions taken to move the patients out of ED (i.e. admitted, discharged, or deceased). Data were entered and cleaned by the designated data entry team at AKU using EpiInfo™ v.3.3.2 (Centers for Disease Control and Prevention, Atlanta, USA) [23]. The analysis was done using SPSS v.19 (IBM corporation, New York, USA) and Stata^® ^v.12 (StataCorp LP, Texas, USA) [24,25].

In the current article, patients with the presenting complaint of "near-drowning" were collectively analyzed. A distinction was made between true near-drowning patients and intentional injury patients with "near-drowning" complaint: true near-drowning patients were those who incurred unintentional near-drowning incidents, whereas the rest were patients who were suicidal or assaulted, but presented to EDs with "near-drowning" complaint. Descriptive analyses were conducted to explore socio-demographic patterns for all patients with "near-drowning" presenting complaints, first collectively and then based on intention. In addition, concomitant injuries and other health conditions were explored to examine hidden reasons for patients to seek care at EDs. Injury-related information and healthcare resource utilization information were also analyzed. For questions allowing multiple responses, results were presented as a summary of outcomes (noted as "Sum" in tables). For instance, there were ten possible dispositions for ED patients: left without being seen, follow-up planned, return if required, referred to other hospital, referred to outside physician, detained for observation, admitted to ward, admitted to intensive care unit, coronary care unit or high dependency unit (ICU/CCU/HDU), expired, or left against medical advice (LAMA). Each patient can have several combinations of the dispositions, such as "detained for observation" combined with "admitted to ward." Although the combination of outcomes are intuitive, they do not directly provide information on different kinds of disposition. Therefore aggregated results were presented as "summary" information in tables. This information represents a proportion of each disposition in relation to one another. In this example, "disposition" was presented as either "discharged" (including discharged from ED, follow-up planned, and return if required) or "admitted" (including admitted to ward, ICU, CCU, HDU, or detained for observation).

## Results

### Intention of presenting complaints recorded as "near-drowning"

Among Pak-NEDS injury patients, 133 patients (0.2% of total injured patients) presented with near-drowning events. Among these 133 patients, intention of injury was recorded for 80, and about half suffered from the injury unintentionally (40 patients, 50.0%). These patients were identified as true near-drowning patients. (Table 1) The other half suffered from intentional injuries, including self-inflicted injuries and assault, which all led to near-drowning complaints. More than a quarter of the near-drowning complaints occurred due to assault (26.3%), whereas nearly one-fourth were self-inflicted (23.7%). (Table 2) Males constitute a higher proportion of intentional injuries that led to near-drowning: 68.4% of self-inflicted cases, 71.4% of assault cases, and 77.5% of unintentional cases.

**Table 1:** Intention of injuries presented as "near-drowning" and the nature of injury

Intention of Injuries	Unintentional (n = 40)	Intentional (n = 40)		Total (n = 80)***
			
	True Near-Drowning (n = 40)	Self-inflicted (n = 19)		
Nature of Injury*	**N (%)**	**N (%)**	**N (%)**	**N (%)**

Sprain/strains/bruises	9 (22.5)	1 (5.3)	4 (19.0)	14 (17.5)
Cut/Open wound	21 (52.5)	11 (57.9)	2 (9.5)	34 (42.5)
Fracture	4 (10.0)	3 (15.8)	5 (23.8)	12 (15.0)
Head injury/contusion	0 (0.0)	1 (5.3)	3 (14.3)	4 (5.0)
Burn**	1 (2.5)	0 (0.0)	1 (4.8)	2 (2.5)
Missing	5 (12.5)	3 (15.8)	6 (28.6)	14 (17.5)

Note: comparing the intent of injury within each nature of injury, no statistical significance was found for any group. (For Sprain/strains/bruises, Cut/open wound, and Fracture, P values were 0.23, 0.07, and 0.21. For Head injury/contusion and burn injuries, chi-square test could not be applied)

* Based on the main nature of injury for each patient, and did not consider other accompanying nature of injury

** Burn: one case of burn<20% (Unintentional), and one case of burn>20% (Assault).

*** Missing: 39.8%

**Table 2:** Socio-demographic characteristics of patients with presenting complaint of "near-drowning"

Characteristics		True Near- Drowning (n = 40)	Intentional Injuries (n = 40)	Total (n = 133)*
		
		N (%)	N (%)	N (%)
*Age Groups*	< 5 years	1 (2.5)	2 (5.0)	3 (2.5)
	5-14 years	2 (5.0)	4 (10.0)	8 (6.7)
	15-24 years	10 (25.0)	14 (35.0)	35 (29.4)
	25-44 years	19 (47.5)	13 (32.5)	49 (41.2)
	45-64 years	6 (15.0)	5 (12.5)	21 (17.6)
	65+ years	0 (0.0)	1 (2.5)	3 (2.5)
*Sex*	Male	31 (77.5)	28 (73.8)	88 (72.1)
	Female	9 (22.5)	12 (30.0)	34 (27.9)

Note: comparing socio-demographic characteristics between unintentional and intentional injuries, no statistical significance was found for any group (for age and sex, P-values were 0.57 and 0.45).

* Missing: 39.8% for intention variable; 10.5% for Age variable; 8.2% for Sex variable.

Taking a closer look at the intention of injuries and their association with the nature of injury, fractures were found to be the most common nature of injury (23.8%) among patients who were nearly drowned due to assault. The percentage of patients presenting with fractures was 15.8% for self-inflicted near-drowning patients and only 10.0% for true near-drowning cases. Assault cases had the highest percentage of head injury/contusions (14.3%), whereas true near-drowning cases were mostly recorded as having cuts or open wounds (52.5%) and sprain/strains or bruises (22.5%). There was no statistical significance in proportions between groups, therefore a 95% confidence interval could not be reported.

### Descriptive epidemiology for patients with presenting complaints of "near-drowning"

Patients aged 25-44 years accounted for the highest proportion who presented with "near-drowning" complaints (n = 49, 41.2%); teenagers and young adults aged 15-24 years contributed to the second highest proportion (n = 35, 29.4%). (Table 2) For true near-drowning cases, 25- to 44-year-olds contributed to the highest proportion (47.5%), whereas intentional injury cases saw the highest proportion among patients aged 15-24 years (35.0%). The proportion of true near-drowning was nearly two times higher among males (77.5%) than females (22.5%).

This study also explored concomitant complaints that these true near-drowning patients and intentional injury patients with "near-drowning" complaint had when visiting the EDs (Table 3). About 21.8% of these patients also had head/face/neck injuries, 22.6% had upper limb injuries, and 29.3% had lower limb injuries (Table 3). Among the 40 true near-drowning patients, 16 (40.0%), 11 (27.5%), and 10 (25.0%) also had lower limb injuries, upper limb injuries, and head/face/neck injuries, respectively. Please note that these conditions are not mutually exclusive, and patients could present with multiple severe conditions. Many other accompanying health conditions were also recorded among the 133 patients, such as loss of consciousness (3.8%), fever (2.3%), and chest pain (1.5%).

**Table 3:** Patients with "near-drowning" presenting complaints with other injuries that required care

Other injuries/conditions* - sum**	True near-drowning patients (n = 40)	Intentional injury patients (n = 40)	Total (n = 133)***
	
	N (%)	N (%)	N (%)
Injury - head/face/neck	10 (25.0)	9 (22.5)	29 (21.8)
Injury - arms/hands	11 (27.5)	7 (17.5)	30 (22.6)
Injury - legs/feet	16 (40.0)	10 (25.0)	39 (29.3)
Injury - Abdomen	0 (0.0)	1 (2.5)	1 (0.8)
Other****	5 (12.5)	6 (15.0)	21 (15.8)

Note: Chi-square test could not be applied because the variable is summary of multiple responses.

* Obtained relevant information from patients' main complaints when presenting at the ED;

** Summary of multiple responses, therefore % provided was for individual category, and cannot add up to 100%

*** Missing: 39.8% for intention variable

**** Other conditions among all 133 patients: Loss of consciousness (5, 3.8%), Fever (3, 2.3%), Chest pain (2, 1.5%), Bleeding with cough (2, 1.5%), Suicidal tendencies (4, 3.0%), Fatigue (n = 1, 0.8%), Fast heartbeat (n = 1, 0.8%), Other heart conditions (n = 1, 0.8%), Pain in abdomen (n = 1, 0.8%), Rectal bleeding (n = 1, 0.8%), Throat pain (n = 2, 1.5%), Wheezing (n = 1, 0.8%), Cough (n = 3, 2.3%), Headache (n = 3,2.3%), Dizziness (n = 1, 0.8%), Anxiety (n = 1, 0.8%), Pain in back (n = 1, 0.8%), Abscess (n = 1, 0.8%), Pain/difficulty in urination (n = 1, 0.8%), General pain (n = 2, 1.5%).

### Emergency department (ED) and hospital resource utilization for near-drowning patients

Only 2.5% of true near-drowning patients arrived at the EDs through an ambulance, and 10.0% of intentional injury patients used an ambulance. Triage was done for 17 patients (15.3%) with "near-drowning" presenting complaint. However, none of the true near-drowning patients received triage assessment. Of the 37 true near-drowning patients among whom disposition information was collected, the majority (85.0%) were discharged home from the ED, including those who had follow-up planned and those who needed to return if required by the care provider. A slightly lower proportion of intentional injury patients were discharged home (80.0%) compared with true near-drowning patients. Medical officers and nurse/midwives provided service to the majority of patients (70.3% and 52.5% patients received care from them, respectively). This observation holds when it comes to true near-drowning patients, with 87.5% receiving care from a medical officer, and 80.0% receiving care from a nurse or midwife. An attending physician only provided care to five patients (4.2% from all whose care provider information was available). Due to missing information for the intention of injury, Table 4 only recorded care provided by two attending physicians - one to a true near-drowning patient, and another to an intentional injury patient. Cardiopulmonary resuscitation (CPR) was provided to 21 patients (17.8%), and a slightly higher proportion of true drowning patients (27.5%) received CPR compared with intentional injury patients (25.0%).

**Table 4:** Emergency Department (ED) Care and Hospital Resource Utilization for Near-Drowning Patients

Care and Resource Information		True near-drowning (n = 40)	Intentional Injuries (n = 40)	Total (n = 133)*
		
		N (%)	N (%)	N (%)
*Mode of Arrival*	Ambulance	1 (2.5)	4 (10.0)	6 (5.0)
	Non-Ambulance	40 (97.5)	32 (80.0)	113 (95.0)
*Type of Visit*	First Visit	39 (97.5)	38 (95.0)	99 (96.1)
	Follow-up Visit	1 (2.5)	2 (5.0)	4 (3.9)
*Triage*	Done	0 (0.0)	6 (15.0)	17 (15.3)
	Not done	35 (87.5)	33 (82.5)	94 (84.7)
Disposition**, ***	Discharged from ED	34 (85.0)	32 (80.0)	87 (81.3)
	Admitted to ward	3 (7.5)	1 (2.5)	15 (14.9)
Type of Care Provider - Sum***	Paramedic	12 (30.0)	29 (72.5)	60 (50.8)
	House officer/intern	15 (37.5)	15 (37.5)	54 (45.8)
	PG trainee/resident	9 (22.5)	6 (15.0)	20 (16.9)
	Medical officer	35 (87.5)	30 (78.0)	83 (70.3)
	Nurse/midwife	32 (80.0)	14 (35.0)	62 (52.5)
	Attending physician	1 (2.5)	1(2.5)	5 (4.2)
Treatment Provided - Sum**, ****	CPR	11 (27.5)	10 (25.0)	21 (17.8)
	Dressing/Debridement	23 (57.5)	21 (52.5)	54 (45.8)
	Antibiotics	17 (42.5)	16 (40.0)	43 (36.4)
	Tetanus toxoid injection	12 (30.0)	14 (35.0)	32 (27.1)
	Painkillers	29 (72.5)	23 (57.5)	88 (74.6)
	Suturing/staples	3 (7.5)	10 (25.0)	14 (11.9)
	Other	18 (45.0)	16 (40.0)	45 (30.1)

Note: Chi-square test could not be applied (for mode of arrival, type of visit and triage, cells with value <5; for disposition, type of care provider and treatment provided, they are summary of multiple responses.)

Abbreviations: ED - Emergency Department; ICU - Intensive Care Unit; CCU - Critical Care Unit; HDU - High Dependency Unit; CT scan - computed tomography scan; CPR - cardiopulmonary resuscitation; I&D - Incision and Drainage of wound; IQR - interquartile range.

* Missing: 39.8% for intention variable; 10.5% for Mode of Arrival variable; 22.6% for Type of Visit variable; 16.5% for Triage variable; 19.5% for Disposition-Combinations variable; 27.8% for Cost of treatment variable.

** Disposition: Discharged - "discharged from ED and follow up planned" or "discharged from ED and return if required"; Admitted to - "admitted to ward", "admitted to ICT/CCU/HDU" or "detained for observation"

*** Summary of multiple responses, therefore % provided was for individual category, and cannot add up to 100%

**** Other types of treatment provided: IV fluids (18, 15.3%), Plaster/cast (15, 12.7%), Ionotropes/pressors (2, 1.7%), ASV & ARV (10, 8.4%).

## Discussion

The current paper analyzed ED-based information in terms of patients with "near-drowning" presenting complaints, depicting socio-demographic patterns, basic injury-related information, concomitant complaints, and preliminary results for the usage of ED resources in seven participating hospitals in Pakistan. The results show the scarcity of drowning and near-drowning data in Pakistan, even when active surveillance was carried out in major EDs across the country. It also highlighted the similarity and differences between true (unintentional) near-drowning events and intentional injuries that led to near-drowning complaints. The article further pointed out potential improvements that are needed in building ED capacity and standardizing data collection systems in this nation.

The results of the Pak-NEDS study have shown similar injury patterns regarding the sex of patients to studies carried out in other LMICs in the region - males were observed to be more prone to injuries compared with females [7,26,27]. However, the distribution of near-drowning patients across age groups revealed a slightly different pattern compared with previous observations. In South Asia, drowning is usually observed among mobile children under the age of five, mostly due to the close proximity to water, inadequate supervision, and the curious nature of young children [9,28]. In the Pak-NEDS results, a higher proportion of near-drowning occurred among the age group of 25-44 years. Nearly 41% of drowning deaths were recorded in the age group of 25 to 44 years, which is the most productive age in Pakistan. This is likely due to the under-reporting of drowning and near-drowning cases, and the potentially biased sample could have reflected an inaccurate picture. For instance, the overall Pak-NEDS study did not capture a substantial number of children, because children might be taken to specialized hospitals that were not included in this study.

The proportion of intentional injuries with "near-drowning" complaint in this study is considerably high, with half of all cases the result of either assault or self-harm [11]. This finding reveals the hidden needs of improving (or establishing) mental health services and proper referral system in Pakistan to prevent suicides and other negative consequences of intentional injuries. Similarly, a previous study from Australia pointed out a significant increase in drowning rates from 1999 to 2002 for which intentional self-harm was the main contributor [29]. Researchers and public health professionals should be more equipped to efficiently and effectively recognize the differences between true drowning or near-drowning cases and intentional injuries that led to drowning or near-drowning. Thus, more research is needed to understand the reasons behind such high rate of intentional injuries. Adequate prevention and management plans should be guided by relevant evidences. Amongst the group of true near-drowning patients that were entered into the current analysis, most presented with cuts or open wounds. Additionally, they presented with common lower-limb injuries. The nature of these injuries might be due to struggling in the water during drowning process. In the current study, fractures were found to be more common among assault victims than true near-drowning patients, so were head injuries and contusions. Head injuries might cause more permanent damage to the lives of patients [30]. Drowning disproportionately affects the more productive individuals in a family. Therefore, if the livelihood of a family usually depends on the injured person, negative impacts can be more chronic and grave [4,7].

The Pak-NEDS study reveals evidence of sub-optimal utilization of emergency medical services (EMS), the most unsatisfying being the 5% utilization rate of emergency vehicles. In 2001, a Pakistan-based study explored the potential explanations for the low utilization rates of emergency vehicles, and concluded that the reasons might include inaccurate recognition of the severity of a patient's condition, unsatisfactory response time of ambulances, inability of some individuals to effectively reach out to an ambulance, as well as unaffordable charge of such services [31]. Although the EMS systems in Pakistan still need drastic improvement, they are going through transformational changes and should be more effectively used in case of emergencies in some participating hospitals, such as the Rescue 1122 Service functioning in the province of Punjab [12].

Further, triage was only done for 15% of patients with "near-drowning" complaints, and none for true near-drowning patients. Accurate triage is known for optimizing resource utilization and facilitating the prevention of secondary injury or even death [32]. Only 21 patients received cardiopulmonary resuscitation (CPR) (half of whom were true near-drowning cases), which is without doubt a necessary step to resuscitate a drowned individual [33]. It is also possible that CPR had been performed by EMS personnel or trained layperson at the scene of injury, prior to or during the transportation of drowning patients. However, we do not have data to support this assumption. After the initial assessment, care received by these patients was mostly analgesia treatment, which may or may not address other potential impacts of the injury, such as respiratory distress syndrome, arrhythmia, sepsis, cerebral edema, fluid overload, and so on [30]. ED care providers are generally advised to reassess the patients' airway, breathing, and circulation (using CPR methods), examine any sign of fracture in the cervical spine (which encourages radiological diagnosis, as was routinely done in EDs in the Pak-NEDS study), monitor oxygen saturation, mental status, and urine output, and pay attention to any sign of hypothermia [8].

One positive observation was that care was generally provided by physicians, nurses, as well as paramedics, which potentially ensures comprehensive care. Interestingly, it is observed in the current study that most of the time junior staff were the first responders when a nearly drowned patient was sent to the ED. Therefore, it is important to train them in the proper management of near-drowning cases. Another relatively reassuring fact was the low hospital admission rate of near-drowning patients, which could be an indicator of high recovery rate of these patients. Nevertheless, this could be a masked result of other undesirable conditions such as limited hospital capacity, lack of in-depth diagnosis, or worse, withdrawal of patients or their families due to financial constraints.

The under-reporting of near-drowning events was possible due to a few combined factors. First, while the burden of drowning is significantly higher in rural areas of Pakistan, all participating EDs in this study were affiliated with urban hospitals, which may not truthfully reflect the true burden at the national level [14]. Second, data collection was carried out from November 2010 to March 2011, which was a low-risk season for drowning events. Summer and monsoon/rainy seasons - typically June until September in Pakistan - are positively associated with drowning rates in South Asia and other regions of the world [7,34-36]. In addition, drowning is usually fatal at the scene of incident and these victims are rarely transported to any health facility, therefore not captured in the ED-based surveillance system [7]. Near-drowning patients, on the other hand, might quickly recover, appearing conscious and functional, and thus may not access ED services. There are other barriers to care-seeking, which have been identified by previous studies, including distance to health facility, financial constraints of low-income households, as well as legal and societal implications for reporting a drowning incident [7]. It has long been reported that ED data by their nature carry some selection bias related to accessibility [37,38]. In the Pak-NEDS study, a high proportion of patients had accompanying injuries of differing degrees of severity. These accompanying conditions may actually be the main causes for these patients to seek medical care instead of the presenting complaints.

Mortality and morbidity caused by drowning and near-drowning are largely neglected in South Asian countries [7]. Half of all drowning-related cases captured by the Pak-NEDS surveillance system were near-drowning only (and the other half were intentional injuries). No drowning cases, i.e. fatal cases, were captured. On one hand, the possible under-reporting of drowning and near-drowning cases limits the opportunity for researchers and policy makers to accurately depict a drowning pattern. On the other hand, this deficit actually provides valuable information regarding the capacity of an ED-based surveillance system to serve as a comprehensive injury registry [12]. Despite the limited capacity of an ED surveillance system to capture drowning and near-drowning cases, it is still feasible and essential to standardize and strengthen these systems for accurate and reliable data collection.

## Limitations

There are a few limitations in the Pak-NEDS study, some of which have been mentioned above, including under-reporting of drowning and near-drowning in EDs; potential selection bias of ED cases; and the impact of seasonal fluctuation on data collection regarding drowning and near-drowning. One additional limitation is the lack of information on the location of near-drowning events, which can potentially guide interventions to prevent unintentional drowning incidents. Overall, it is important for researchers to note the limitations of ED-based data, which is reflected by the under-reporting of drowning-related incidents in this study. There will also be additional bias due to the higher severity of diseases seen at EDs compared with other departments in a hospital. This factor highly depends on the care-seeking pattern of patients in different countries and regions. It is also revealed that follow-up visits were notably low, which is true for the majority of ED cases reported elsewhere [39]. The difficulty in managing subsequent follow-ups creates additional barrier to study the chronic outcomes of injuries and other diseases.

## Conclusion

The Pak-NEDS study is a valuable reminder for researchers to carefully assess the information they need, and measure it against existing data collection systems. Community and household-level data are more preferable in the case of drowning events; however, these kinds of data collection activities can be extremely time consuming and resource intensive, and can hardly exceed the scale of a nationwide ED-based surveillance system. In order to control for temporality or seasonality, subsequent ED-based surveillance should cover a full seasonal cycle--especially for drowning and near-drowning events. As is made evident by many high-income countries, the negative impact of drowning on health systems can be largely reduced through collective actions across multiple sectors - a wide range of strategies can be borrowed from existing HIC preventive programs and modified to suit LMIC settings [6,33,40-42]. The Pak-NEDS results are the essential first step in grasping the scope of the drowning burden in Pakistan; uncovering the shockingly close correlation between violence, intentional self-harm and drowning; pinpointing weak links in study implementation; and setting priorities for future collaborative interventions in drowning prevention in Pakistan and beyond.

## Competing interests

The authors declare that they have no competing interests.

## Authors' contributions

SH and JCL developed the initial draft. Data management and analysis was done by SH. NZ, URK and KS critically reviewed the draft. AAH designed and supervised the Pak-NEDS study, and critically reviewed the draft. All authors approved the final draft.
